# Planning ability impairments in probable Alzheimer's disease patients: Evidence from the Tower of London test

**DOI:** 10.1590/1980-57642016dn11-020006

**Published:** 2017

**Authors:** Corina Satler, Luiza Guimarães, Carlos Tomaz

**Affiliations:** 1 PhD, Adjunct Professor, Faculty of Ceilandia, UnB, University of Brasilia, Brasilia, DF, Brazil; 2 Undergraduate Student, Laboratory of Neurosciences and Behavior, Department of Physiological Sciences, University of Brasilia, Brasilia, DF, Brazil; 3 PhD, Full Professor, Neuroscience Research Program, University CEUMA, São Luis, MA, Brazil

**Keywords:** cognition, dementia, elderly, executive function, neuropsychology, cognição, demência, idoso, função executiva, neuropsicologia

## Abstract

Alzheimer's disease (AD) is associated with progressive impairment of higher-level cognitive abilities. Previous research suggests that early impairment of executive functions occurs during the course of the disease, but few studies have specifically investigated planning ability in an AD population.

**Objective:**

The purpose of the current study was to examine whether AD patients retain the ability to plan ahead, by analyzing specificities of their behavior in successfully achieving a pre-established goal.

**Methods:**

Twenty-one AD patients and thirty-three elderly controls underwent a problem-solving assessment using the Tower of London (TOL) test.

**Results:**

AD patients were less accurate and less efficient than controls. AD patients also committed more mistakes. This indicates a decline in working memory and inhibitory deficits, resulting in impulsive and inappropriate behaviors.

**Conclusion:**

These results are in agreement with previous studies, showing executive function problems in patients with AD. Specifically, this study demonstrates the presence of planning ability deficits in AD, considering both qualitative and quantitative approaches. The wide range of analysis presented in this study can aid clinicians in identifying the nature of the poor performance of AD patients during a planning task.

## Abstract (pt)

A doença de Alzheimer (DA) é associada a um comprometimento progressivo das habilidades cognitivas superiores. Pesquisas anteriores sugerem que o comprometimento precoce das funções executivas ocorre durante o curso da doença, mas poucos estudos têm investigado especificamente a capacidade de planejamento em uma população com DA.

**Objetivo:**

O objetivo do presente estudo foi analisar se os pacientes com DA mantêm a capacidade de planejar antecipadamente, analisando as especificidades de seu comportamento para alcançar com êxito uma meta pré-estabelecida.

**Métodos:**

Vinte e um pacientes com DA e trinta e três controles idosos foram submetidos a uma avaliação de resolução de problemas utilizando o teste de Torre de Londres (TOL).

**Resultados:**

Os pacientes com DA foram menos precisos e menos eficientes do que os controles. Os pacientes com DA também cometeram mais erros. Isso indica um declínio na memória operacional e déficits inibitórios, resultando em comportamentos impulsivos e inadequados.

**Conclusão:**

Estes resultados estão de acordo com estudos anteriores, mostrando problemas de função executiva em pacientes com DA. Especificamente, este estudo demonstra a presença de déficits de capacidade de planejamento na DA, considerando abordagens qualitativas e quantitativas. A ampla gama de análises apresentadas neste estudo poderá auxiliar aos profissionais da área da saúde na identificação da natureza do baixo desempenho dos pacientes com DA durante uma tarefa de planejamento.

## INTRODUCTION

Alzheimer's disease (AD) is the most common cause of dementia among adults aged over 65 years. AD is a slowly progressive neurodegenerative process, with typically insidious onset, characterized by neuronal atrophy, synapse loss, and abnormal deposition of B-amyloid protein plaques and neurofibrillary tangles within specific regions of the brain.^1^ According to the pattern of neuropathological changes associated with progression to AD, the earliest changes occur in medial temporal lobe limbic structures. This process gradually spreads to affect temporal, frontal and parietal lobes.^2,3^

Consistent with these alterations, higher-level cognitive abilities are affected early in the course of AD. Episodic memory impairment is usually the earliest and most salient aspect of the AD dementia syndrome. Additionally, deficits in attention, language and visuospatial abilities, processing speed, and executive functions (EF) may be present from the beginning of the illness.^1,4,5^ Given this profile, executive dysfunction can be considered a common manifestation during the course of AD, occurring at all stages of the disease.^1,6-9^

The term "executive functions" refers to various complex cognitive processes and sub-processes that are thought to control or guide behaviors in a top-down manner. There is general agreement that EF encompass three main components: inhibition, working memory, and cognitive flexibility. From these, a set of higher order cognitive processes are built, such as reasoning, problem solving, and planning.^10^ These processes enable us to formulate goals and plans; remember these goals over time; choose and initiate actions to help us achieve these goals; and monitor and adjust our behavior, as necessary, until we complete or fail at them.^11^

Evidence from neuropsychological studies shows that the prefrontal cortex plays an important role in EF. As early as 1868, J. M. Harlow affirmed that frontal-lobe lesions in humans result in a loss of "planning skills".^12^ Several decades later, Bianchi (1922) described how monkeys with large frontal lesions were unable to coordinate different elements of a complex activity.^12^

Planning and future-directed behavior involve a variety of aspects of EF, including plan formulation, monitoring and regulation of the responses intended to carry out the plan, the capacity to maintain goal representations in working memory, inhibition of attention to distracting stimuli, and sustained suppression of impulsive response.^12,13^

Neuropsychological tests based on tower paradigms have been used as a reliable measure of planning and problem solving abilities. The Tower of London (TOL) test was developed by Shallice and McCarthy^14^ as an alternative to the classic Tower of Hanoi test.

The TOL test is considered a complex planning task that relies on multiple executive operations including inhibitory control, set maintenance, cognitive flexibility, self-monitoring, working memory, and attention allocation.^12,13^ It is assumed that the solution of the test is best accomplished by the use of strategy and by planning a sequence of moves without breaking the predefined rules.^15^

Given the TOL test's widespread use in clinical and research settings, there are several versions that differ with regard to whether they are computerized or standard, the number of attempts, and scoring criteria. The test has been suggested as a useful tool for neuropsychological examination of healthy and clinical populations. Planning impairments on the TOL test have been observed among both acute^16,17^ and chronic neurological disorders,^18-20^ as well as in psychiatric conditions.^21,22^

Additionally, TOL performance has been associated with instrumental activities of daily living.^23,24^ Planning ability is thought to be important to "real world" activities,^25^ so an executive dysfunction may hinder the performance of simple everyday tasks, such as brushing teeth, cooking, or shopping.^26^

Everyday action errors are frequent in various clinical groups and may impair performance in achievement of the task goal.^27,28^ Progressive inability to perform activities of daily living is one of the diagnostic criteria for AD,^29^ and leads to a loss of independence, affects the patient's quality of life, and increases the burden of caregivers by shifting many daily responsibilities to them.^30^ This feature of the disease is closely associated with the above-mentioned cognitive decline.^1,4,5^

The prediction that AD patients will show poor performance during EF tests is supported by an abundance of neuropsychological test findings.^1,4,8,31^

Substantial progress has been made in determining the extent of planning deficits in AD. Nevertheless, unlike the numerous studies focusing on the TOL scoring system for AD diagnosis and use of the tool for neuropsychological examinations of patients with mild cognitive impairment,^20,32-35^ only a few studies^35,36^ have included a qualitative description based on observation of AD patient performance in their investigation of TOL performance.

The aim of this study was to examine whether probable AD patients retain the ability to plan ahead, with particular attention to the specificity of their responses for successfully achieving a pre-set goal. Considering that the TOL test involves the ability to generate and execute a successful sequence of moves while anticipating and avoiding incorrect moves, and based on earlier findings, we expected that AD patients would show significantly poorer performances compared to normal controls, as well as impairments in processing speed, inhibitory control, and working memory. Additionally, we expected the presence of perseverations and *closing-in behavior*" (CB) for AD patients, considering that CB is the expression of a default sensorimotor organization normally inhibited by executive control,^37^ and that this inhibition can break down under conditions of reduced executive resources, as is the case with AD.

## METHODS

The study included 21 patients with a diagnosis of probable AD and 33 healthy elderly adults (EC).

All AD patients met the AD criteria described in the DMS-IV (ed.4), published by the American Psychiatric Association in 1994, and defined by the NINCDS-ADRDA. A clinical diagnosis of probable AD was determined for each patient at an interdisciplinary team meeting (including a social worker, a neuropsychologist, and a geriatrician). The severity of AD ranged from mild to moderate according to the Clinical Dementia Rating Scale (CDR).^38^ Patients exhibited a history of progressive cognitive impairment, which was confirmed by their caregiver using the Informant Questionnaire on Cognitive Decline in the Elderly (IQCODE).^39^ All subjects underwent a neuropsychological evaluation (Table 1). This study was approved by the Human Subject Committee of the Faculty of Medicine (FM-UnB).

**Table 1:** Demographic and neuropsychological data for Alzheimer' disease patients and elderly controls.

	Alzheimer' disease M (SD)	Elderly controls M (SD)
Age, years Range	78.90 (6.16)*68-88	70.81 (7.07)61-84
Sex (female/male)	16/5	21/12
Education, years Range	7.04 (3.84)*4-15	11.84 (4.37)4-22
Duration of illness, years	3.73 (1.77)	0
Clinical Dementia Rating score	1.28 (0.46)*	0
Functional Activities Questionnaire score	19.57 (6.62)*	0.09 (0.52)
IQCODE score	3.87 (0.58)*	2.79 (0.50)
Neuropsychiatric Inventory score	16.19 (10.54)*	5.18 (5.55)
CSDD score	10.04 (5.53)**	5.90 (4.25)
Mattis Dementia Rating (144 max)	111.57 (8.48)*	139.75 (3.68)
Mini-Mental State Examination (30 max)	17.71 (4.13)*	28.39 (1.45)
Clock Drawing Test - command (10 max)	4.71 (2.90)*	9.00 (1.90)
Clock Drawing Test - copy (10 max)	7.47 (2.54)*	9.72 (0.57)
Phonemic fluency - FAS	16.90 (10.01)*	36.12 (13.48)
Semantic fluency - Animals	5.57 (2.74)*	17.03 (4.60)

IQCODE: Informant Questionnaire on Cognitive Decline in the Elderly. CSDD: Cornell Scale for Depression in Dementia. Values expressed as mean (SD). Significant differences comparing AD with EC group using t-test are indicated as follows:

* p < .001.

** p < .05.

**Procedure.** Subjects were tested individually in a room with normal interior lighting at the Geriatric Medical Center at the University Hospital of Brasilia, Brasilia.

The Krikorian version of the TOL test^15^ was used in this study, because of its suitability for use with dementia patients.^20^ Subjects were instructed to transform the start configuration into the target configuration while following three rules: (1) they had to reproduce the examiner's model in a minimum number of moves; (2) only one ball may be moved at a time; (3) a ball may not be placed on the table or held in the hand while another ball was being moved. We also simplified the rule concerning placement of the balls in relation to the length of the pegs.^36^

When rules were broken, subjects were asked to restart the problem. Subjects were also asked to restart the problem if a wrong final configuration was presented.

The stopwatch was started when the two configurations were revealed to the subject and started again when the subject made the first move.

**Scoring.** Accuracy of the problem solution was analyzed based on the number of attempts needed to achieve the correct final configuration for each problem, taking into consideration the prescribed minimum number of moves (raw score of 0 to 36 or 0 to 100 percent).^15^ The terms "accuracy" and "success" are henceforth used interchangeably.

Two time measures were utilized to analyze each problem: "*initiation time*" (IT), the time from the presentation of a test problem by the examiner to the initiation of the first problem-solving move by the subject, and "*execution time*" (ET), the time from initiation of the first move to completion or discontinuation of problem-solving.

In accordance with Rainville et al.,^36^ we analyzed three different types of errors: (1) "*wrong final configuration*"-WFC; (2) "r*ule breaking*"-RuleB; and (3) "*excess movements*"-EM.

We analyzed five complementary behaviors: (1) "*interrupted move*"-IM is when the subject started the action and then stopped, such as when they plainly lifted the ball from the peg and then held it in the air; (2) "*hesitation behavior*"-HB refers to the action of touching or almost touching the ball, without removing it completely from the peg; (3) "*regret behavior*"-RB occurs when the subject moved a ball from a peg, and then placed it back on the same peg that it had been on before starting the move; (4) "*perseverative behavior*"-PB refers to inappropriate maintenance of a sequence of movements in an effort to solve the problem; and (5) "*closing-in behavior*"-CB refers to the action of trying to move a ball from the examiner's tower, instead of the subject's own tower.

**Statistical methods.** Descriptive and inferential statistical analyses were performed to characterize the sample. We compared the AD and EC groups with regard to their problem-solving accuracy for the entire set of problems, their time performance, errors, and complementary behaviors. A multivariate analysis of covariance (MANCOVA) was performed with the group as the independent factor, and age and education as covariates. We also conducted an ANOVA of repeated-measures, with group as the independent factor, and age and education as covariates in order to compare the accuracy of the solutions to the problems across the four levels of the test and five moves. We then conducted a new t-test for independent samples, this time for each problem individually.

Partial correlations (*pr*), maintaining both age and education constants, were used to assess the relationship between the TOL measures for AD patients. We also conducted a *pr* to examine a possible association between the presence of closing-in behaviors and dementia severity (CDR), functional activities of daily living (FAQ), and global cognitive status (MMSE and DRS total scores). Analyses were performed using the PSAW Statistics software (v.18.0 for Windows). The level of statistical significance was set at 5% (p<.05).

## RESULTS

Frequency analysis showed that that 38.1% of the AD sample failed to achieve a total score above 50% success, and only seven patients (33.3%) were capable of performing the 12 problems successfully, in contrast to the ECs, who successfully completed all the problems.

The MANCOVA yielded a significant between-group difference, reflecting the better performance by the EC group, *F*(11, 39)=6.50, *p* < .001, η^2^=.64. Post hoc contrast revealed that the AD patients were less accurate, needed more time to perform the test, exhibited more errors and performed more complementary behaviors than ECs during the test. The AD group showed a lower success score, needed more time to execute the test, broke a greater number of rules, made more WFCs, hesitated more, made more interruptions, had more regrets, and performed more closing-in. The two groups did not differ with regard to IT, EMs, and PBs (see Table 2).

**Table 2:** Performance analysis for Alzheimer' disease patients (AD) and elderly controls (EC).

	Group					F(1,49)	p	η^2^	f^2^	95%CIs
	**AD**			**EC**						
	**M**	**(SD)**		**M**	**(SD)**					
Total success score	21.50	10.08		34.12	1.89	27.67	.000	.36	.99	27.67
Initiation time	200.75	118.92		151.06	112.74	.72	.398	.01	.13	.72
Execution time	440.06	223.85		241.19	76.10	11.18	.002	.18	.90	11.18
Excess movements	4.30	3.41		2.45	2.62	2.44	.124	.04	.33	2.44
Rule breaking	9.92	6.28		1.96	2.33	22.50	.000	.31	.99	22.50
Wrong final configuration	5.25	4.24		1.90	1.99	5.29	.026	.09	.61	5.29
Hesitation behavior	42.11	44.05		18.18	10.93	8.72	.005	.15	.82	8.72
Interrupted move	10.38	7.87		5.17	3.94	14.70	.000	.23	.96	14.70
Regret behavior	9.25	8.33		3.51	2.98	11.50	.001	.19	.91	11.50
Perseverative behavior	1.31	2.33		.03	.21	1.61	.210	.03	.23	1.61
Closing-in behavior	6.43	7.20		.113	.50	17.47	.000	.26	.98	17.47

Mean and standard deviation are raw scores.

**Figure 1:**
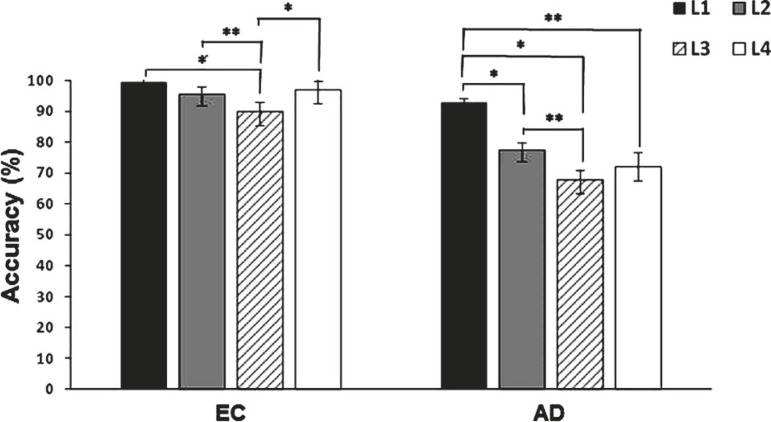
Mean success score (%) in each level of the TOL test for Alzheimer’s disease patients (AD) and elderly controls (EC). Significant differences within groups are indicated as follows: *p < .001; **p < .05.

Analyses of accuracy across the four levels of the test showed a significant correlation between the level and the group, *F*(3, 41)=4.66, *p*=.007, η^2^=.25. Within-subject analyses revealed a significant contrast (*p*<.001) between level 1 and 2, level 1 and 3, level 1 and 4, level 2 and 3, but not between level 2 and 4 (*p*=.472) nor level 3 and 4 (*p*=.623) for AD patients. The EC group showed significant differences between the four levels of the test (*p*<.035), except between level 1 and 2 (*p*=.088), level 1 and 4 (*p*=.057), and level 2 and 4 (*p*=.557) (Figure 1).

**Figure 2:**
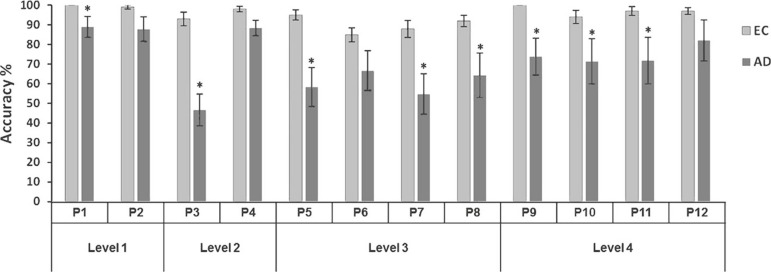
Mean total success score (%) in each problem of the TOL test for Alzheimer’s disease patients (AD) and elderly controls (EC). Significant differences between groups using the Student-*t* test are indicated as follows: *p < .001.

Analyses of individual problems revealed significant differences between groups (*p*<.050), except for problems 2 (*p*=.081), 4 (*p*=.136), 6 (*p*=.106), and 12 (*p*=.182). AD patients obtained a higher percentage of success for the easiest problems when compared to the more difficult problems. In problems 1, 2 and 4, which require only 2 or 3 moves, they obtained close to 90% success, and only a 46.6% success rate for problem 3 (see Figure 2). The EC group obtained a success rate of 91-100% for most problems, except problems 6 and 7.

### Correlations for TOL performance scores of AD patients.

Results showed a significant correlation between the success score and EMs (*p*<.001). There was a significant negative correlation between RuleB (*p*=.040) and CB (*p*=.003) (Table 3). As expected, there was a significant correlation between IT and ET (*p*<.001). Regarding analysis of errors, there was a significant correlation between RuleB and WFC (*p*=.006), IM (*p*<.001), and CB measurements (*p*<.001). Complementary behavior measurements identified significant correlations (*p*<.001) between the following: HB and IM; HB and RB; HB and CB; IM and RB, RB and PB, and RB and CB.

**Table 3:** Partial correlations for TOL performance scores of patients with Alzheimer's disease.

Measures	TOL	IT	ET	EM	RuleB	WFC	HB	IM	RB	PB
TOL										
IT	-.06									
ET	-.12	.62*								
EM	.51*	.00	-.06							
RuleB	-.28**	.21	.41*	-.15						
WFC	-.20	.50*	.65*	-.20	.38*					
HB	-.13	.56*	.81*	-.02	.22	.50*				
IM	-.22	.49*	.71*	.09	.51*	.45*	.69*			
RB	-.21	.52*	.78*	-.12	.46*	.41*	.77*	.51*		
PB	-.08	.15	.37*	-.08	.10	.60*	.05	.16	.36**	
CB	-.40*	.13	.43*	-.17	.44*	.32**	.33**	.24	.38*	.20

TOL: Tower of London success score. IT: Initiation Time score. ET: Execution Time score. EM: Excess Movements score. RuleB: Rule Breaking score. WFC: Wrong Final Configuration score. HB: Hesitation Behavior score. IM: Interrupted Move score. RB: Regret Behavior score. PB: Perseverative Behavior score. CB: Closing-in behavior score.

* *p* < .005.

** *p* < .05.

Finally, results showed a significant correlation (*p*<.001) between closing-in behavior and CDR, FAQ, and a significant negative correlation with MMSE (*p*=.045) and DRS (*p*<.001) (Table 4).

**Table 4:** Partial correlations for closing-in behaviors of patients with Alzheimer's disease.

Measures	Closing-in B	CDR	FAQ	MMSE	DRS
Closing-in B					
CDR	.44*				
FAQ	.52*	.94*			
MMSE	-.27**	-.87*	-.85*		
DRS	-.50*	-.87*	-.87*	.86*	-

Closing-in B: Closing-in behavior score. CDR: Clinical Dementia Rating. FAQ: Functional Activities Questionnaire. MMSE: Mini-Mental State Examination total score. DRS: Mattis Dementia Rating total score.

* p < .005.

** p < .05.

## DISCUSSION

The purpose of this study was to examine whether AD patients retain the ability to plan ahead, by analyzing specificities of their behavior in successfully achieving a pre-set goal. We hypothesized that this ability would be relatively well retained in ECs, as opposed to AD patients. In line with previous evidence,^20,36^ we found that ECs outperformed AD patients, indicating that planning ability is diminished in these patients.

Success rates were lower in AD patients compared to ECs. Although AD patients obtained a high accuracy for some problems, they failed to find the correct solution on the first attempt for most problems, indicating difficulties in generating and executing a sequence of moves to achieve a predetermined goal. AD patients had the greatest trouble solving problem 3. This problem is the first that involves an intermediate move and anticipatory load, so it seems that this problem relies on more complex mental strategies than the first two problems.

The TOL test is a complex planning task, which relies on multiple cognitive processes involving a variety of EF aspects.^12-15^ Our results suggest that these higher-level cognitive skills are diminished in AD patients. This is bolstered by the observation that AD patients have several cognitive impairments even in very early stages of the disease,^1,5^ with the presence of executive difficulties being common.^4,8^

We also observed time differences between the groups during the test. Although age-related changes in processing speed are expected,^40^ AD patients showed significantly higher time-related measurements than ECs. These results are in line with evidence that AD patients suffer an abnormal decline in mental processing speed.^5^ Owen et al.^41^ examining a group of patients with frontal lobe lesions, described slower performance compared to controls, which was associated with inefficient preplanning and the need for extra on-line planning during the execution of the test.

For problem 12, AD patients had the longest ET and an 80% success rate, suggesting more time with a better performance, contrary to problem 3, where a longer execution time and a low success score was observed. Taking the success score and time-related measurements into account, our findings therefore disagree with Krikorian et al.^15^ who claimed that levels 1 and 2 could be solved through a perceptual strategy. This reinforces growing evidence suggesting that problem difficulty is not restricted only to the number of required movements,^42,43^ and that some problems with few moves exert complex cognitive demands.

Findings for WFC showed that AD patients needed more attempts than ECs to achieve the correct final model, with an average of five attempts throughout the test.

Research has emphasized the role of inhibition on performance during the TOL test,^12,36^ since the task includes specific rules that must be adhered to in finding the solution. Although we simplified the rules in our study, AD patients committed an average of seven prohibited actions during the test, which may be associated with a decline in working memory and inhibitory control, including self-monitoring of planning efforts.

AD patients performed more complementary behaviors than ECs. The overuse of IM, hesitation, and RBs in the former may be associated with the presence of goalsubgoal conflict resolution difficulties. This lack of planning is based on the fact that an early incorrect move can make the problem virtually unsolvable, thereby requiring a step back and a new plan on how to achieve the correct solution. These high scores suggest difficulties in mentally storing and manipulating information over short periods, and may additionally be associated with the use of trial and error.

Perseverations and CBs were also seen in AD patients. During the TOL test, this phenomenon was exhibited by AD patients who failed to switch to a new configuration, repeating the same responses from a preceding configuration. Sandson and Albert^44^ categorized this perseverative behavior as "stuck-in-set", proposing that it involves an underlying deficit of EF. This type of behavior is frequently demonstrated during various cognitive tests, indicating impairment of inhibitory processes.^31^

Regarding CBs, our results are consistent with other studies, which have described this phenomenon in AD patients.^37,45^ We highlight the ''attraction hypothesis'',^46^ which describes this phenomenon as a reflection of the disinhibition of a primitive behavior (automatic tendency). Additionally, as suggested by other researchers,^45^ this behavior was noticeable in our clinical sample and showed a significant positive correlation with dementia severity (CDR).

Several conclusions can be drawn from this research: (1) Our findings are consistent with previous studies showing that AD patients had difficulties in planning ahead and executing complex predetermined plans; (2) The difficulties encountered when taking the TOL test are not restricted to the number of required movements; (3) Reducing the number of rules did not improve AD patient performance; (4) The TOL test was able to distinguish AD patients from ECs, considering both qualitative and quantitative approaches. We recommend further studies that include complementary analyses, which can aid clinicians in identifying the nature of the poor performance of AD patients; (5) Specific behaviors (RuleB and CB) exhibited by AD patients are useful indicators of impairments in inhibitory processes. Finally, the TOL test provides clinically relevant information that can be used to augment treatment planning and care, prioritizing well-being and quality of life of AD patients.

Further studies involving larger sample groups are necessary to confirm these results.
